# Artificial intelligence for prediction of clinical response and therapeutic value in interventional pain management: a scoping review

**DOI:** 10.3389/fdgth.2026.1833918

**Published:** 2026-07-02

**Authors:** Mariana González Garcés, Jerónimo Cárdenas Montoya, Valeria Concha Fernández, Mario Andrés Torres Torres, Erwin Hernando Hernández Rincón

**Affiliations:** 1 School of Medicine, Universidad de La Sabana, Chía, Colombia; 2 Department of Family Medicine and Public Health, Universidad de La Sabana, Chía, Colombia

**Keywords:** artificial intelligence, interventional pain management, machine learning, predictive models, value-based care

## Abstract

**Introduction:**

Interventional pain management is characterised by substantial variability in clinical response, durability of benefit and risk of adverse events, which limits traditional decision-making approaches based on empirical procedure selection. In this context, artificial intelligence has been increasingly explored as a methodological approach to examine predictive strategies and value-oriented decision frameworks in complex interventional settings.

**Objective:**

To map and characterise the available scientific evidence on the application of artificial intelligence techniques for predicting clinical response, procedural risk and dimensions of therapeutic value in adult patients undergoing interventional pain procedures.

**Methods:**

A scoping review was conducted in accordance with the Joanna Briggs Institute methodology and reported following the PRISMA-ScR guidelines. A systematic search was performed in PubMed, Scopus, Web of Science and IEEE Xplore for studies published between 2015 and 2026. Eligible studies applied artificial intelligence or machine learning models to explore outcome prediction in interventional pain management.

**Results:**

Twenty-five studies were included. Most investigations examined predictive applications in epidural injections, radiofrequency procedures, vertebral augmentation and spinal cord stimulation. Across these domains, artificial intelligence models were used to explore patterns associated with clinical response, durability of benefit and procedural risk. Additional outcome domains included opioid use trajectories, functional recovery and identification of scenarios associated with potentially low therapeutic value. The majority of studies were retrospective in design and relied primarily on internal validation, with limited external validation reported.

**Conclusions:**

The available evidence indicates that artificial intelligence has been applied across multiple interventional pain domains to explore predictive approaches related to clinical response and therapeutic value. However, methodological heterogeneity, retrospective study designs and limited external validation restrict the interpretability and clinical transferability of these findings. Further prospective studies with robust external validation are required before routine clinical implementation can be considered.

**Systematic Review Registration:**

https://osf.io/a8esc/.

## Introduction

Chronic pain represents one of the most prevalent and disabling health conditions worldwide, exerting a substantial impact on physical functioning, quality of life and healthcare system sustainability. According to recent estimates from the Global Burden of Disease, chronic pain syndromes, particularly those of musculoskeletal and neuropathic origin, consistently rank among the leading causes of years lived with disability across all regions, with an increasing trend driven by population ageing and improved survival with chronic disease (1–3). Beyond its individual clinical burden, chronic pain generates significant economic consequences related to recurrent healthcare utilisation, productivity loss and long-term functional dependence.

In response to this growing burden, pain management has progressively evolved towards more specialised therapeutic strategies. Interventional pain management has assumed a central role for patients with pain refractory to conservative treatments. Procedures such as epidural injections, image guided nerve blocks, radiofrequency ablation and neuromodulation techniques have been increasingly incorporated into clinical practice with the aim of achieving analgesia, improving function and reducing persistent symptom burden (4–6).

Despite their expanding use, a uniform understanding of the sustained clinical benefit and therapeutic value of interventional pain procedures in routine practice remains lacking. Population based studies have demonstrated marked variability in utilisation rates across regions, institutions and individual clinicians, exceeding what would be expected from epidemiological or clinical differences alone (7–9). This variability has been interpreted as an indirect signal of coexisting patterns of overuse, underuse and inappropriate use, with relevant implications for care quality and health system efficiency.

Within interventional pain management, therapeutic value can be operationally defined as a multidimensional construct encompassing clinical response, durability of benefit, risk of adverse events, functional outcomes, patient reported benefit and the efficient use of healthcare resources. Interventions of low therapeutic value therefore correspond to those providing marginal, inconsistent or short-lived benefit, particularly when associated with procedural risk or disproportionate resource utilisation (10–12).

From a public health perspective, low value care represents a substantial challenge. A significant proportion of healthcare expenditure in high income countries has been attributed to practices that do not deliver benefits proportional to their costs, especially in technology intensive fields (13, 14). In this context, value-based care frameworks emphasise the need to optimise intervention selection by aligning clinically meaningful patient outcomes with responsible resource allocation (15).

International initiatives aimed at reducing unnecessary practices, such as Choosing Wisely, have promoted evidence informed decision making and limitation of inappropriate procedures (16). However, implementation within interventional pain management remains challenging. Pain heterogeneity, subjective symptom perception, comorbidities and pronounced interindividual variability in treatment response limit the applicability of uniform criteria derived solely from clinical guidelines or randomised trials (17, 18).

In this complex clinical environment, traditional approaches are often insufficient to accurately identify patients most likely to benefit from a given intervention or those at higher risk of poor response. Artificial intelligence (AI) has emerged as a methodological approach with the potential to address this challenge by enabling integrated analysis of large scale clinical, procedural and longitudinal data, and by identifying complex response patterns not readily captured through conventional statistical methods (19–21).

Across multiple medical domains, machine learning (ML) and deep learning (DL) models have been applied to support clinical decision making, outcome prediction and reduction of unwarranted practice variation (22–24). In interventional pain management, these techniques offer the opportunity to refine patient selection, anticipate treatment response and contribute to the avoidance of low value interventions by integrating clinical variables, imaging data, procedural characteristics and patient reported outcomes (25, 26).

Despite this potential, the existing evidence on AI applications for predicting clinical response and therapeutic value in interventional pain management remains fragmented and methodologically heterogeneous. To date, no structured synthesis has comprehensively mapped the scope, characteristics and limitations of published studies in this field. A scoping review therefore represents the most appropriate methodological approach to describe the current evidence landscape, identify recurring patterns and highlight knowledge gaps (27, 28).

Accordingly, the objective of this scoping review is to map and characterise the existing evidence on the application of AI for predicting clinical response and evaluating therapeutic value of interventional pain procedures in adult patients with chronic pain, with particular emphasis on its potential to support clinical decision making and reduce low value care.

## Methods

### Study design

A scoping review was conducted to map and characterise the available scientific evidence on the application of AI techniques for predicting clinical response, identifying non-responders and estimating therapeutic value in adult patients undergoing interventional pain procedures. This design was selected due to its suitability for synthesising heterogeneous evidence and describing emerging patterns of application in complex clinical contexts.

The review was conducted in accordance with the Joanna Briggs Institute methodological guidance and is reported following the PRISMA ScR recommendations. The study protocol was prospectively registered in the Open Science Framework and is available at https://osf.io/a8esc.

As this study was designed as a scoping review, no formal assessment of risk of bias or methodological quality of the included studies was performed.

### Research question and PCC framework

The research question was structured using the Population, Concept and Context framework.

The population included adult patients with chronic or persistent pain undergoing interventional pain procedures, predominantly of musculoskeletal or neuropathic origin.

The concept comprised the application of AI techniques, including ML and DL algorithms, aimed at predicting clinical response, identifying non-response or therapeutic failure, and estimating dimensions of therapeutic value relevant to clinical decision making.

The context included specialised interventional pain services in hospital-based and outpatient settings.

The research question was defined as follows:

What scientific evidence exists on the use of AI techniques to predict clinical response and therapeutic value of interventional pain procedures in adult patients with chronic pain?

### Search strategy and data sources

A comprehensive literature search was performed in PubMed, Scopus, Web of Science and IEEE Xplore. Studies published between January 2015 and January 2026 in English or Spanish were eligible.

The lower temporal limit was set at 2015, reflecting a methodological shift towards the application of modern AI techniques to real-world clinical data. The upper limit corresponds to the date of the final search and ensured inclusion of the most recent evidence.

The complete search strategies for each database are reported in Table 1. A manual snowball search of reference lists was also conducted. All records were managed using Rayyan.

**Table 1:** Search strategies.

Database	Search algorithm
PubMed	((“Artificial Intelligence”[Mesh] OR “Machine Learning”[Mesh] OR “Deep Learning”[Mesh] OR artificial intelligence [tiab] OR machine learning [tiab] OR deep learning [tiab] OR algorithm*[tiab] OR decision support [tiab] OR clinical decision support [tiab]) AND (“Pain Management”[Mesh] OR “ Chronic Pain”[Mesh] OR interventional pain [tiab] OR interventional pain management [tiab] OR pain intervention*[tiab] OR nerve block*[tiab] OR epidural injection*[tiab] OR radiofrequency [tiab] OR neuromodulation [tiab] OR spinal cord stimulation [tiab] OR facet joint [tiab]))
Web of Science	TS = (“artificial intelligence” OR “machine learning” OR “deep learning” OR “predictive model*” OR “ risk prediction” OR “ clinical prediction”) And TS = (“interventional pain” OR “interventional pain management” OR “pain intervention*” OR “nerve block*” OR “epidural injection*” OR radiofrequency OR neuromodulation OR “spinal cord stimulation”) And TS = (chronic OR persistent OR neuropathic)
SCOPUS	TITLE-ABS-KEY(“artificial intelligence” OR “machine learning” OR “deep learning” OR “predictive model” OR “risk prediction” OR “clinical prediction”) AND TITLE-ABS-KEY(“interventional pain” OR “interventional pain management” OR “pain intervention” OR “nerve block” OR “epidural injection” OR radiofrequency OR neuromodulation OR “spinal cord stimulation”) AND TITLE-ABS-KEY(chronic OR persistent OR neuropathic)
IEEE Xplore	(“artificial intelligence” OR “machine learning” OR “deep learning” OR “predictive model” OR “clinical prediction”) And (pain OR analgesia) And (intervention OR procedure OR injection OR block OR neuromodulation OR radiofrequency)

### Study selection

**Figure 1:**
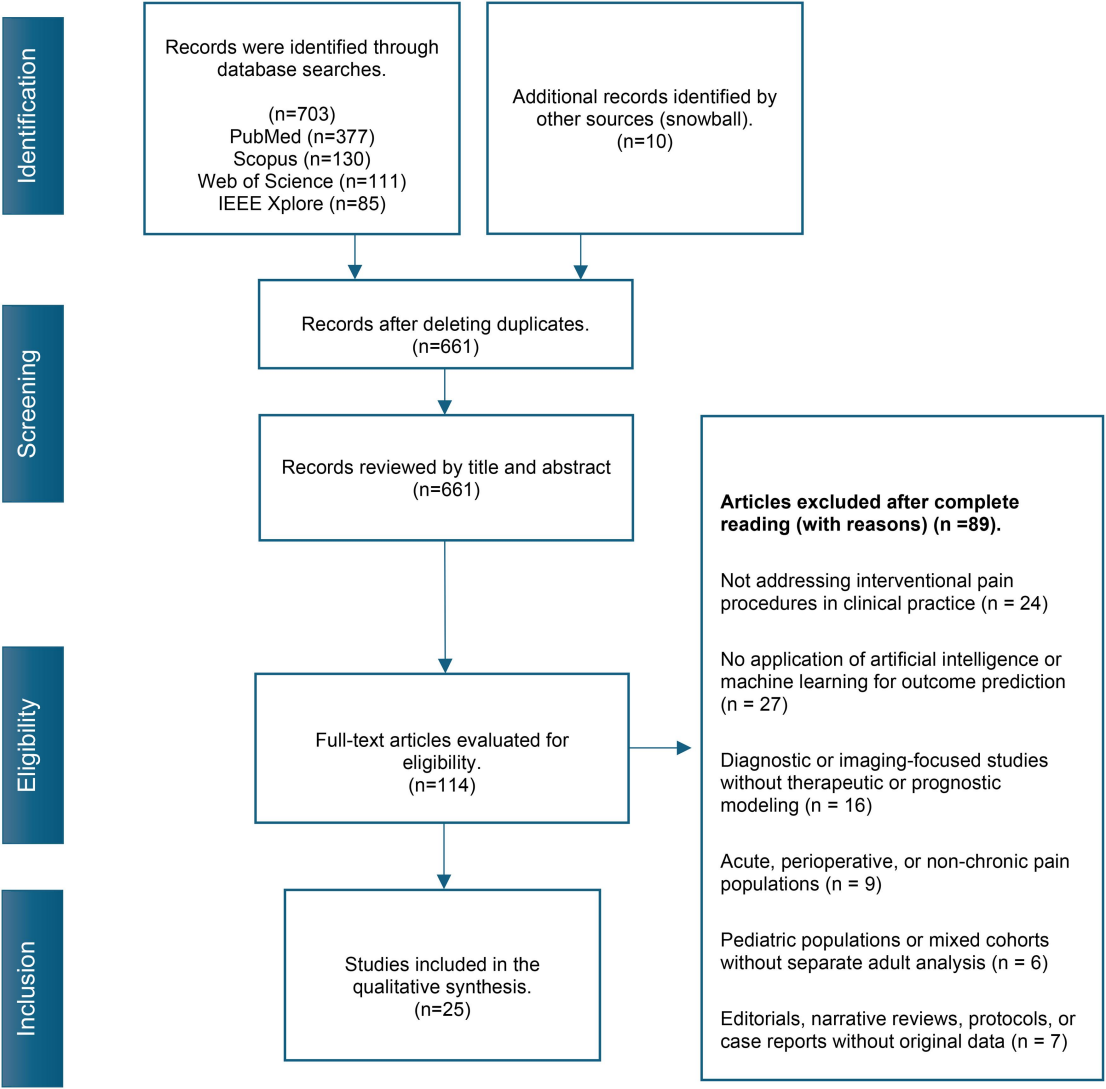
PRISMA-ScR flow diagram of the study selection process.

After duplicate removal, records were independently screened by two reviewers at title and abstract level. Studies meeting preliminary eligibility criteria underwent full-text assessment to confirm alignment with the predefined PCC framework. The selection process is summarised in the PRISMA ScR flow diagram (Figure 1).

### Data extraction, analysis and synthesis

Data were extracted using a structured Microsoft Excel matrix. Extracted variables included study design, sample size, population characteristics, type of interventional procedure, AI model objective, algorithm type, data sources, predicted outcomes, performance metrics and validation strategies. Reported AI approaches included ML, DL and radiomics-based models. Extracted performance metrics included area under the receiver operating characteristic curve (AUC), accuracy, sensitivity, specificity, F1-score, calibration-related measures and discrimination metrics when available. Validation strategies were classified as internal (split-sample validation, cross-validation or temporal validation within the same dataset) or external (independent cohort validation).

For the purposes of synthesis, the variables that directly informed the analytical framework were the type of interventional procedure, the predicted clinical outcome, the dimension of therapeutic value addressed, the model objective and the validation approach. The remaining extracted variables were used primarily for descriptive characterisation of the included studies.

A narrative synthesis was conducted, grouping studies according to intervention type, predicted outcome and therapeutic value dimension, with particular emphasis on identification of potential responders, non-responders and implications for low-value care. Given the methodological heterogeneity across included studies, quantitative pooling or meta-analysis was not considered appropriate.

## Results

### Overall structure of the identified evidence

The integrated analysis of the included studies indicates that AI applications in interventional pain management do not constitute a homogeneous body of evidence, but rather a field organised across distinct domains with different levels of clinical maturity. These domains arise from the interaction between procedure type, predicted outcome and the specific dimension of therapeutic value addressed, allowing differentiation between applications with immediate clinical applicability and those still confined to exploratory settings.

Based on this synthesis, three main domains were identified: applications focused on prediction of therapeutic response, applications centred on procedural risk stratification and safety, and emerging applications aimed at technical optimisation or clinical decision support. The distribution and characteristics of these domains are summarised in Tables 2, 3.

**Table 2:** Predictive performance patterns of artificial intelligence across interventional pain applications.

AI application domain	Primary predicted outcome	Representative predictive performance	Validation characteristics	Key performance pattern	References
Epidural steroid injections	Analgesic response	High discriminative performance in imaging-based models	Predominantly internal validation	Imaging-based models consistently outperformed clinical-only models	(29–31)
Facet joint radiofrequency	Short- and long-term pain relief	Consistent prediction of short- and long-term outcomes	Temporal validation reported in selected studies	Interpretable ML enabled identification of stable prognostic factors	(32, 33)
Trigeminal neuralgia interventions	Treatment response	Moderate-to-high radiomic discrimination capacity	Internal validation predominated	Radiomics-derived features showed strong discriminative capacity	(34–36)
Vertebral augmentation procedures	Fracture, refracture, complications	Robust risk stratification performance	Predominantly internally validated CT-based models	CT-based radiomics achieved robust risk stratification	(37–40)
Neuromodulation (SCS)	Sustained pain relief	Moderate-to-high long-term predictive performance	Limited external or multicenter validation	Multimodal models showed superior long-term prediction	(41–45)
Opioid-related outcomes post-SCS	Dose reduction or stabilization	Moderate predictive performance	Internal validation	ML models predicted opioid trajectories beyond pain scores	(46)
Procedural targeting and planning	Anatomical accuracy	High segmentation accuracy	Internal validation	Deep learning segmentation improved procedural precision	(47)
Acute care triage (LLM-based)	Decision guidance	Exploratory feasibility-level performance	Exploratory validation only	LLMs demonstrated feasibility but limited clinical validation	(48)

AI, artificial intelligence; ML, machine learning; MRI, magnetic resonance imaging; CT, computed tomography; SCS, spinal cord stimulation. Predictive performance descriptions are presented narratively to summarize heterogeneous reporting across included studies and should not be interpreted as pooled quantitative estimates.

**Table 3:** Outcome domains successfully predicted by artificial intelligence in interventional pain management.

Outcome domain	Direction of prediction	Evidence profile	Clinical implication	References
Therapeutic response	Responder vs non-responder	Consistent	Enables pre-procedural patient selection	(29, 30, 32–36, 41–45)
Risk of complications or failure	Increased vs. reduced risk	Consistent	Supports individualized risk–benefit assessment	(37–40)
Durability of benefit	Sustained vs. transient response	Moderate	Facilitates long-term treatment planning	(32, 42, 44, 45, 49)
Structural disease progression	Progression vs. stability	Moderate	Guides surveillance strategies	(40, 49)
Functional and satisfaction outcomes	Improvement vs. no improvement	Moderate	Complements pain intensity metrics	(31, 50, 51)
Opioid trajectory	Reduction or stabilization	Emerging	Expands value beyond pain relief	(46)
Procedural precision	Accurate vs. suboptimal targeting	Consistent	Enhances technical success	(47)
Clinical decision support	Appropriate vs. delayed action	Exploratory	Potential role in acute pain pathways	(48)

Evidence profiles are presented descriptively to summarize heterogeneous predictive performance and validation characteristics across included studies.

**Figure 2:**
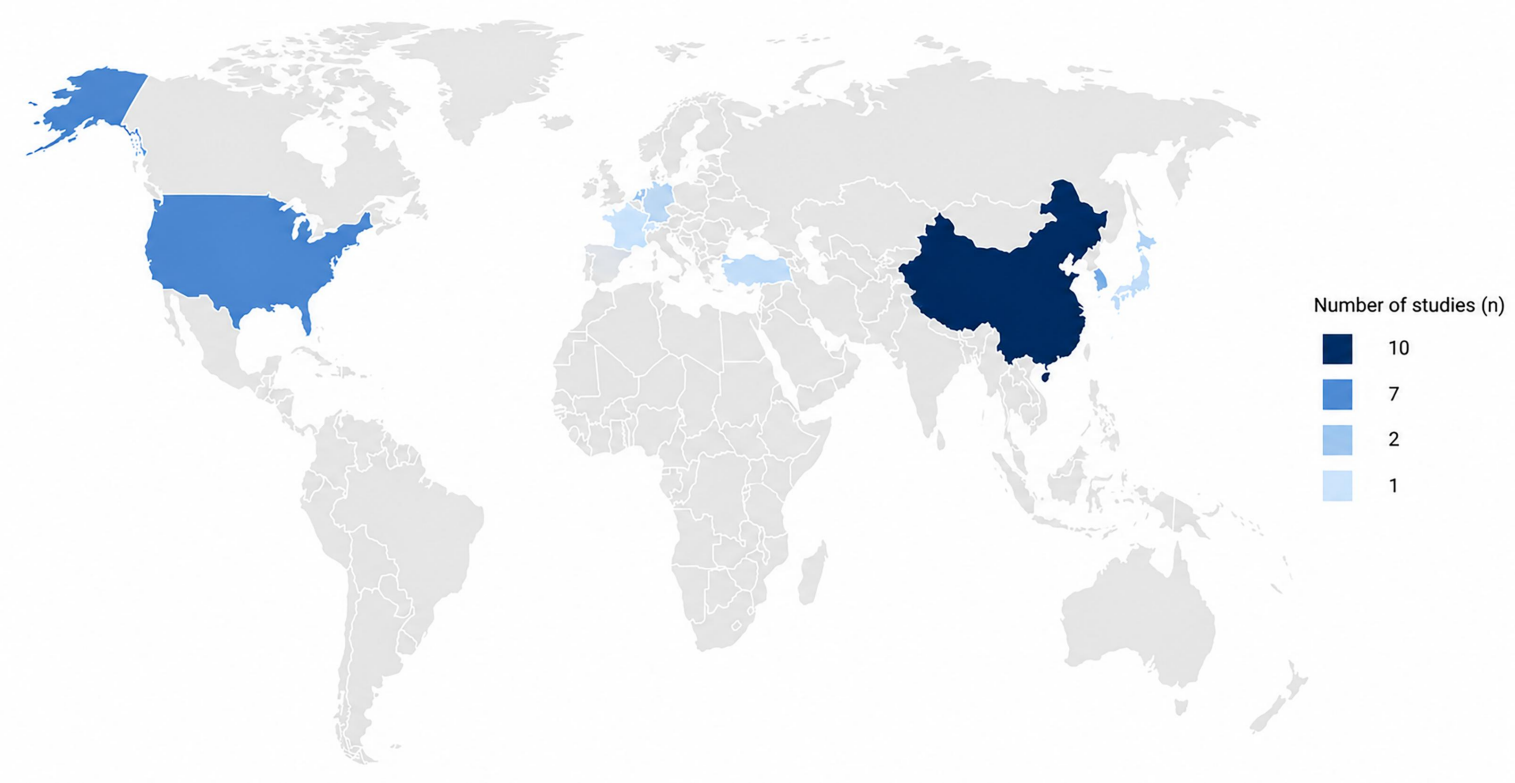
Geographic distribution of the included studies based on the primary institutional affiliation of the coordinating authors. China contributed the highest number of studies (*n* = 10), followed by the United States (*n* = 7). South Korea and the Netherlands each contributed two studies (*n* = 2), whereas Switzerland, Belgium, France, and Turkey each contributed one study (*n* = 1). The base map was adapted from Natural Earth (https://www.naturalearthdata.com/), Public Domain.

From a geographical perspective, the evidence was concentrated primarily in Asia, North America and Europe, with a predominance of studies conducted in China and the United States. This distribution reflects the dependence of AI applications on access to large scale clinical datasets, advanced imaging platforms and adequate computational infrastructure, as illustrated in Figure 2.

In summary, the field presents a structured but uneven evidence landscape, with clearly identifiable domains at different stages of clinical readiness.

Across the included studies, the most frequently applied AI approaches included convolutional neural networks, random forest models, support vector machines, gradient boosting algorithms and radiomics-based ML frameworks. Data sources varied substantially and included magnetic resonance imaging (MRI), computed tomography (CT), clinical variables, neurophysiological data, wearable device outputs and patient-reported outcome measures. Reported predictive performance metrics were heterogeneous and included area under the receiver operating characteristic curve (AUC), accuracy, sensitivity, specificity and discrimination analyses. Several studies reported high discriminative performance, particularly in imaging-based prediction models and vertebral risk stratification applications, whereas outcome prediction in neuromodulation and opioid trajectory studies demonstrated greater variability. Validation strategies were predominantly internal, including split-sample and cross-validation approaches, while only a limited number of studies incorporated temporal or external validation. Detailed characteristics of representative studies reporting explicit AI model characteristics, predictive performance metrics and validation strategies are presented in Supplementary Table 1.

### Domain I: prediction of therapeutic response

Prediction of clinical response represented the most mature and extensively studied domain of AI application in interventional pain management. This domain accounted for the largest proportion of included studies and demonstrated the most consistent predictive patterns, particularly in procedures with well-defined anatomical correlates.

In cervical and lumbar transforaminal epidural injections, DL models trained on MRI data were used to estimate analgesic response, suggesting that imaging-based features capture clinically relevant anatomical and degenerative information not systematically incorporated into conventional clinical assessment (29, 30). These findings position epidural procedures as priority candidates for AI assisted patient selection strategies.

In radiofrequency procedures for lumbar facet mediated pain, prediction extended beyond initial analgesic response to include differentiation between transient and sustained benefit. Several studies modelled the temporal durability of response, enabling estimation of longer-term therapeutic benefit prior to intervention (32, 33).

In trigeminal neuralgia, radiomic models derived from MRI identified morphological patterns associated with treatment response. However, greater methodological variability was observed across studies, indicating that further standardisation of imaging acquisition and analytical pipelines is required before broader clinical translation (34–36).

Overall, prediction of therapeutic response constitutes the core domain of the field, with the highest level of consistency and proximity to potential clinical implementation.

### Domain II: procedural risk stratification and safety

A second clearly differentiated domain comprised applications of AI for procedural risk stratification and prediction of adverse events, particularly in vertebral interventions. In this domain, the analytical focus shifted from analgesic efficacy towards safety and prevention of structural complications.

Computed tomography-based models were applied to predict adjacent level fracture, refracture and vertebral recompression following vertebroplasty or kyphoplasty. These outcomes redefine therapeutic value in vertebral procedures by prioritising complication avoidance and long-term structural integrity over short term symptom relief (37–40).

Given the direct implications of these predicted outcomes for procedural decision making and patient safety, this domain represents one of the areas with the highest potential readiness for clinical translation.

In summary, risk stratification applications expand the concept of therapeutic value by incorporating safety and harm prevention as central outcome dimensions.

### Domain III: neuromodulation and personalised outcome prediction

Neuromodulation, particularly spinal cord stimulation, represented one of the most methodologically complex domains identified. AI models integrated clinical, neurophysiological and longitudinal data to predict sustained response to treatment, offering an alternative to empirical selection strategies based on temporary stimulation trials (41–45).

Several studies extended outcome prediction beyond pain intensity by modelling trajectories of opioid reduction or stabilisation following spinal cord stimulation. This approach introduces a broader conceptualisation of therapeutic value that encompasses clinical, economic and public health relevant outcomes (46).

This domain illustrates a transition from symptom centred prediction towards personalised therapeutic trajectories informed by multidimensional data.

### Emerging applications and exploratory domains

A smaller subset of studies addressed emerging applications that remain exploratory but provide early signals of innovation. DL based anatomical segmentation models were applied to improve procedural planning accuracy in image guided interventions, with potential implications for technical reproducibility and procedural safety (47).

In parallel, large language models were explored as decision support tools in acute pain settings. While technical feasibility was demonstrated, the current level of evidence remains insufficient to support systematic clinical adoption (48).

These exploratory applications highlight future research directions but remain limited by early stage validation.

### Cross domain synthesis of outcomes

Across domains, the predicted outcomes reflect an expansion of what constitutes a clinically relevant result in interventional pain management. Prediction of therapeutic response remains the central application, followed by procedural risk stratification and estimation of benefit durability. Outcomes related to structural progression, functional status and opioid use trajectories represent emerging areas with potential clinical impact (31, 46, 49–51).

Taken together, the results position AI in interventional pain management as a field in transition, characterised by the coexistence of applications approaching clinical implementation and others requiring further external validation and methodological standardisation. The structured organisation of evidence presented in Tables 2, 3 provides a framework to guide future research priorities and clinical adoption strategies.

## Discussion

The findings of this scoping review should be interpreted as exploratory and hypothesis-generating. They describe how AI has been studied within interventional pain management, rather than providing evidence to support direct clinical decision-making or routine clinical implementation.

### Contextualisation of the findings within interventional pain management

Interventional pain management has long been characterised by substantial uncertainty in predicting individual patient outcomes. Even when procedures are performed according to accepted technical standards and guideline-based indications, interindividual variability in clinical response and durability of benefit remains considerable. Prior clinical literature has consistently shown that a significant proportion of patients undergoing epidural injections, radiofrequency procedures or neuromodulation experience limited, transient or clinically insufficient benefit, often accompanied by repeated interventions and persistent symptoms (52–54).

The studies included in this review describe how AI has been explored as an analytical approach to address this uncertainty. By integrating clinical variables, imaging-derived features and longitudinal data, these models aim to estimate expected outcomes before an intervention is performed. Importantly, this body of work does not indicate that AI replaces clinical reasoning, but rather reflects ongoing efforts to better characterise variability in treatment response within complex clinical settings.

### Relationship with traditional approaches to patient selection

Conventional patient selection strategies in interventional pain management rely primarily on clinical assessment, diagnostic testing and classical statistical models. Existing evidence has demonstrated that these approaches have limited capacity to reliably distinguish sustained responders from patients with partial or absent benefit, particularly in spinal and facet-based interventions (9, 54).

The studies mapped in this review report the use of ML and DL models to explore non-linear associations between patient characteristics, imaging findings and outcomes. These approaches should be understood as complementary analytical tools that may enhance understanding of response patterns, rather than as replacements for established clinical evaluation. Their role remains investigational, and their outputs require careful interpretation within the broader clinical context.

### Procedure-specific heterogeneity in AI applications

The results indicate that the application of AI varies substantially across interventional procedures. In cervical and lumbar transforaminal epidural injections, imaging-based models trained on MRI data were frequently used to explore prediction of therapeutic response. These applications appear particularly suited to conditions where anatomical and degenerative features are closely linked to procedural mechanisms (29–31).

In lumbar facet radiofrequency procedures, several studies extended outcome modelling beyond initial analgesic response to include estimation of response durability. This temporal dimension is clinically relevant, as the duration of benefit influences long-term management strategies and cumulative procedural exposure. Nevertheless, these findings should be interpreted as descriptive of model performance within specific datasets, rather than as evidence supporting routine predictive use (32, 33).

### Vertebral procedures and emphasis on safety-related outcomes

In vertebral augmentation procedures, AI applications focused predominantly on risk stratification rather than on analgesic efficacy. Models based on CT data were used to estimate the likelihood of adjacent vertebral fracture, refracture and vertebral recompression (37–40).

This shift in analytical focus reflects an expanded conceptualisation of therapeutic value that incorporates safety and harm prevention. Traditional risk assessment approaches in this context have relied largely on clinical and densitometric variables and have shown limited predictive capacity for structural complications (55, 56). The findings identified in this review suggest that AI has been explored as a means of addressing these limitations, although its clinical impact remains to be demonstrated.

### Neuromodulation and reassessment of empirical selection paradigms

Neuromodulation, particularly spinal cord stimulation, represents one of the most methodologically complex domains identified. Historically, candidate selection has relied on temporary stimulation trials, whose ability to predict sustained clinical benefit has been questioned in prior non-artificial intelligence-based literature (57–60).

The studies included in this review describe AI models that integrate clinical, neurophysiological and longitudinal data to explore prediction of long-term response (41–45). A limited number of studies also examined opioid use trajectories following spinal cord stimulation, introducing an outcome domain with potential clinical and health system relevance (46, 59). These findings should be regarded as exploratory and do not support changes to existing selection paradigms without further validation.

### Broadening of clinically relevant outcome domains

An important observation emerging from this review is the progressive broadening of outcome domains considered relevant in interventional pain management. While traditional literature has prioritised pain intensity as the principal indicator of treatment success, the included studies examined additional outcomes such as durability of benefit, structural disease progression, functional status, patient satisfaction and opioid use trajectories (31, 49–51).

This multidimensional perspective aligns with contemporary critiques of relying solely on pain intensity measures to assess therapeutic effectiveness (6, 25). However, heterogeneity in outcome definitions, measurement tools and reporting standards remains a major barrier to synthesis and comparison across studies.

### Clinical and organisational considerations

From a clinical perspective, the findings of this review suggest that AI has been investigated as a potential tool to support more nuanced understanding of patient heterogeneity and expected treatment trajectories. Early identification of patients at higher risk of non-response may inform consideration of alternative therapeutic pathways, although this remains a theoretical implication rather than an evidence-based recommendation.

At an organisational level, predictive models may have implications for resource utilisation and reduction of low-value care, particularly in areas characterised by high procedural variability (10, 12, 15). These implications remain speculative and require evaluation through prospective implementation studies.

### Limitations of the current evidence base

Despite growing interest, the available evidence presents important methodological limitations. Most included studies were retrospective and relied predominantly on internal validation strategies, limiting generalisability and real-world clinical applicability. Substantial heterogeneity was observed across study design, data sources, outcome definitions, AI methodologies, reported performance metrics and validation frameworks, precluding direct quantitative comparison across studies.

In addition, relatively few studies incorporated prospective patient-reported outcome measures or external validation cohorts, both of which are critical for evaluating clinical utility and implementation readiness. The geographical concentration of studies in a limited number of countries, particularly China and the United States, also raises concerns regarding potential algorithmic bias, dataset shift and limited transferability across healthcare systems and patient populations.

As this review was designed as a scoping review intended to map the available evidence landscape, no formal risk of bias assessment was performed. Accordingly, the findings should be interpreted as exploratory and hypothesis-generating rather than as evidence supporting immediate clinical implementation.

Additional challenges include limited model interpretability, integration into real-world clinical workflows and ethical governance. AI should therefore currently be regarded as a decision-support research tool rather than a substitute for clinical judgement.

### Future directions

The findings of this scoping review highlight the need for prospective, multicentre studies with external validation and standardised outcome frameworks. Future research should assess not only predictive performance, but also the real-world impact of AI on clinical decision-making, patient-centred outcomes and reduction of low-value interventions.

Greater methodological standardisation will also be necessary to improve reproducibility and comparability across studies. Future primary studies should consider adherence to emerging artificial intelligence-specific reporting and implementation frameworks, including TRIPOD-AI and DECIDE-AI, particularly for prediction model development, validation and clinical deployment.

**Figure 3:**
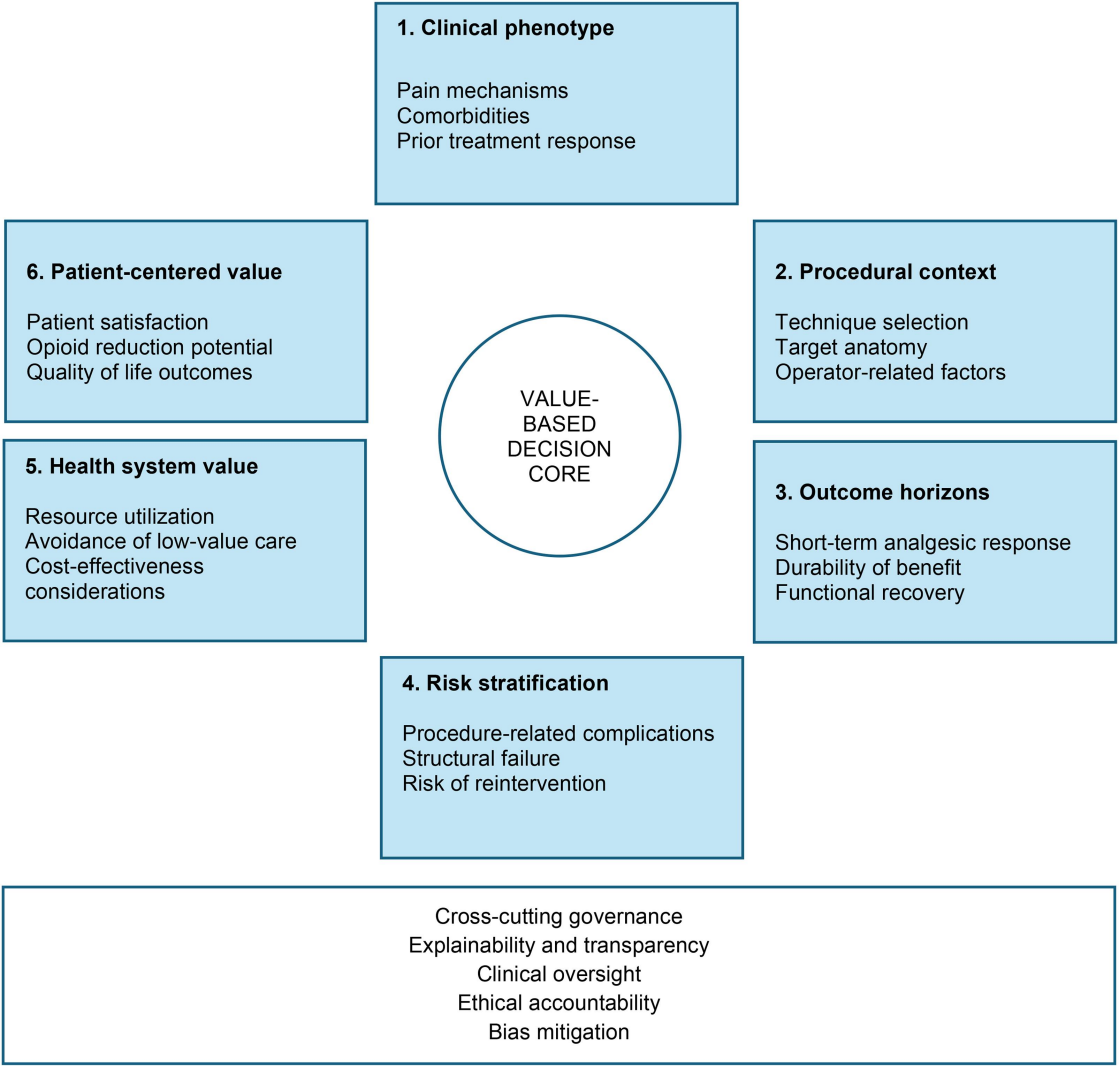
Multidimensional framework for AI-driven value assessment in interventional pain management. The framework depicts how artificial intelligence integrates clinical phenotype, procedural context, outcome horizons, risk stratification, patient-centered value, and health system value into a centralized value-based decision core. Cross-cutting governance principles, including explainability, ethical accountability, and clinical oversight, support responsible implementation of AI-assisted decision making.

Figure 3 illustrates this evolving conceptual framework, depicting a transition from empirically driven procedural decision-making towards exploratory, data-informed approaches that integrate algorithmic outputs with clinical expertise and patient preferences.

## Conclusions

The evidence analysed in this scoping review indicates that AI has been increasingly applied within interventional pain management to explore approaches that shift attention from procedural execution alone towards the anticipatory estimation of clinical benefit. The included studies describe how predictive models have been used to estimate individual probabilities of therapeutic response, durability of benefit and procedural risk prior to intervention, highlighting limitations of traditional trial-and-error strategies.

These developments have potential implications for clinical practice. The ability to characterise patients with a higher likelihood of benefit and, equally importantly, to identify scenarios associated with low expected therapeutic value suggests that AI may contribute to more informed procedure selection, reduction of unnecessary reinterventions and improved efficiency within healthcare systems. Within this framework, therapeutic value extends beyond immediate analgesic relief to encompass functional outcomes, procedural safety and patient-centred results.

Nevertheless, translation into routine clinical practice requires caution. Methodological heterogeneity, limited external validation and challenges related to model generalisability and integration into real-world clinical workflows remain significant barriers. The future role of AI in interventional pain management is therefore likely to depend not solely on increasingly complex algorithms, but on the development of transparent, ethically robust and value-oriented clinical frameworks in which algorithmic predictions support, rather than replace, expert clinical judgement.

## Data Availability

The original contributions presented in the study are included in the article/Supplementary Material, further inquiries can be directed to the corresponding author.
