# Supplementary material for: Artificial intelligence for prediction of clinical response and therapeutic value in interventional pain management: a scoping review
Source: Front Digit Health. 2026 Jul 2;8:1833918. doi: 10.3389/fdgth.2026.1833918 (PMC13373859; doi:10.3389/fdgth.2026.1833918)
Supplement: Supplementary file 1 [file Table1.docx]

**SUPPLEMENTARY MATERIAL**

**Supplementary Table 1. Representative artificial intelligence studies reporting predictive performance metrics and validation strategies in interventional pain management**

| **Domain I. Prediction of therapeutic response** | | | | |
| --- | --- | --- | --- | --- |
| **Study** | **AI approach** | **Predicted outcome** | **Representative performance metrics** | **Validation strategy** |
| **Wang et al. 2023 (29)** | Deep learning CNN using MRI | Analgesic response | AUC ~0.80–0.84 | Internal split validation |
| **Kim et al. 2022 (30)** | Deep learning MRI-based model | Pain reduction response | AUC ~0.83 | Internal validation |
| **Wang et al. 2025 (33)** | Interpretable machine learning with SHAP analysis | Short- and long-term pain relief | AUC ~0.80–0.88 | Temporal validation |
| **Wu et al. 2024 (35)** | Radiomics-based machine learning | Treatment response | AUC ~0.80 | Internal validation |
| **Mulford et al. 2022 (36)** | Radiomics model | Identification of symptomatic trigeminal nerves | AUC ~0.72–0.78 | Internal validation |
| **Wu et al. 2024 (37)** | Radiomics-based ML prediction model | Procedural response | AUC ~0.86 | Internal validation |
| **Domain II. Procedural risk stratification and safety** | | | | |
| **Study** | **AI approach** | **Predicted outcome** | **Representative performance metrics** | **Validation strategy** |
| **Fan et al. 2019 (31)** | Deep learning segmentation model | Anatomical segmentation accuracy | Dice coefficient >0.80 | Internal validation |
| **Yu et al. 2024 (38)** | Nomogram-based predictive model | Adjacent segment disease | C-index and calibration analysis | Internal validation |
| **Yang et al. 2025 (39)** | CT radiomics ML model | Adjacent vertebral fracture | AUC ~0.90 | Internal validation |
| **Zhao et al. 2024 (40)** | Multiple machine learning models | Vertebral adverse events | AUC ~0.81–0.89 | Internal validation |
| **Zhao et al. 2025 (41)** | Logistic regression-based predictive model | Vertebral recompression | AUC ~0.80 | Internal validation |
| **Shen et al. 2024 (42)** | Imaging-based predictive model | Adjacent vertebral refracture | AUC ~0.84 | Internal validation |
| **Domain III. Neuromodulation and personalised outcome prediction** | | | | |
| **Study** | **AI approach** | **Predicted outcome** | **Representative performance metrics** | **Validation strategy** |
| **Hadanny et al. 2022 (43)** | Machine learning prediction models | Sustained treatment response | Accuracy and AUC reported | Internal validation |
| **Gopal et al. 2025 (44)** | ML neurophysiological prediction model | Surgical outcome prediction | ROC and discrimination analyses | Internal validation |
| **Goudman et al. 2020 (46)** | Machine learning techniques | Response to SCS | AUC ~0.74–0.82 | Internal validation |
| **Witjes et al. 2025 (47)** | ML using magnetoencephalography data | Chronic pain/SCS response classification | Classification accuracy reported | Internal validation |
| **Adil et al. 2022 (49)** | Clinical machine learning model | Opioid dose reduction/stabilization | AUC ~0.75 | Internal validation |
| **Ounajim et al. 2021 (51)** | Machine learning algorithms | Response to spinal cord stimulation | Predictive superiority over screening trial | Multicenter validation |
| **Exploratory and emerging applications** | | | | |
| **Study** | **AI approach** | **Predicted outcome** | **Representative performance metrics** | **Validation strategy** |
| **Wang et al. 2025 (34)** | Large language models | Decision guidance and acute pain triage | AUC ~0.80–0.88 | Exploratory validation |
| Abbreviations: AI, artificial intelligence; ML, machine learning; CNN, convolutional neural network; MRI, magnetic resonance imaging; CT, computed tomography; AUC, area under the receiver operating characteristic curve; ROC, receiver operating characteristic; SHAP, Shapley additive explanations. Supplementary Table 1 summarizes representative studies reporting explicit predictive performance metrics and validation strategies across heterogeneous AI applications in interventional pain management. | | | | |
